# Could Stress Contribute to Pain-Related Fear in Chronic Pain?

**DOI:** 10.3389/fnbeh.2015.00340

**Published:** 2015-12-17

**Authors:** Sigrid Elsenbruch, Oliver T. Wolf

**Affiliations:** ^1^Institute of Medical Psychology and Behavioral Immunobiology, University Hospital Essen, University of Duisburg-EssenEssen, Germany; ^2^Department of Cognitive Psychology, Institute of Cognitive Neuroscience, Faculty of Psychology, Ruhr University BochumBochum, Germany

**Keywords:** chronic pain, pain-related fear, Pavlovian conditioning, extinction, memory, stress

## Abstract

Learning to predict pain based on internal or external cues constitutes a fundamental and highly adaptive process aimed at self-protection. Pain-related fear is an essential component of this response, which is formed by associative and instrumental learning processes. In chronic pain, pain-related fear may become maladaptive, drive avoidance behaviors and contribute to symptom chronicity. Pavlovian fear conditioning has proven fruitful to elucidate associative learning and extinction involving aversive stimuli, including pain, but studies in chronic pain remain scarce. Stress demonstrably exerts differential effects on emotional learning and memory processes, but this has not been transferred to pain-related fear. Within this perspective, we propose that stress could contribute to impaired pain-related associative learning and extinction processes and call for interdisciplinary research. Specifically, we suggest to test the hypotheses that: (1) extinction-related phenomena inducing a re-activation of maladaptive pain-related fear (e.g., reinstatement, renewal) likely occur in everyday life of chronic pain patients and may alter pain processing, impair perceptual discrimination and favor overgeneralization; (2) acute stress prior to or during acquisition of pain-related fear may facilitate the formation and/or consolidation of pain-related fear memories; (3) stress during or after extinction may impair extinction efficacy resulting in greater reinstatement or context-dependent renewal of pain-related fear; and (4) these effects could be amplified by chronic stress due to early adversity and/or psychiatric comorbidity such depression or anxiety in patients with chronic pain.

## Introduction and Scope

Pain is a ubiquitous and uniquely aversive experience with strong emotional components. As such, unavoidable pain is universally feared and literally “hard to forget”. Indeed, virtually every one of us can readily recall previous painful episodes, even if they occurred years or decades ago, typically motivating strong avoidance behavior driven by pain-related fear. Importantly, pain-related memories are not limited to sensory-discriminative information such type and duration of pain but also include emotional responses as well as information about the entire context surrounding the painful episode. This is due to the fact that acute pain is more than merely a sensory experience. It rather evokes a range of reactions encompassing complex cognitive, emotional, motivational and motor components that are ultimately aimed at self-protection (Lumley et al., 2011). This set of responses is centrally coordinated within the brain, and involves multiple interconnected bodily systems including afferent sensory pathways and efferent effector systems including the hypothalamus-pituitary-adrenal (HPA) axis and the sympathetic nervous system (SNS). As such, from an evolutionary perspective, the complex response to acute pain constitutes a highly adaptive, fundamental response that is preserved across species. Fear of pain is an essential component of this adaptive response, which however may come to be maladaptive in chronic pain (Vlaeyen, 2015). Pain-related learning and memory processes envoked by recurrent painful experiences induce complex emotional and behavioral changes which likely contribute to the pathophysiology of chronic pain (Flor, 2012). From a learning perspective, associative as well as instrumental learning and memory processes play a crucial role (Flor, 2012; Gatzounis et al., 2012; Vlaeyen, 2015) and are probably intricately intertwined involving both interoceptive and exteroceptive conditioning. We are only beginning to understand how fear of pain is acquired and extinguished. Within this perspective, we attempt to integrate evidence on associative learning and memory processes from the fields of stress and transfer it to pain. We propose that stress may contribute to impaired pain-related learning and extinction processes and thereby play a role in the transition from acute to chronic pain and/or the maintenance of chronic symptoms. In the case of extinction learning and extinction retrieval, the fascinating question arises whether stress influences the original acquisition memory trace and/or the later developed inhibitory extinction memory trace. Specifically, we propose that: (1) extinction-related phenomena inducing a re-activation of maladaptive pain-related fear (e.g., reinstatement, renewal) likely occur in everyday life of chronic pain patients and may alter pain processing, impair perceptual discrimination and favor overgeneralization; (2) acute stress prior to or during acquisition of pain-related fear may facilitate the formation and/or consolidation of pain-related fear memories; (3) stress during or after extinction may impair extinction efficacy resulting in greater reinstatement or context-dependent renewal of pain-related fear; and (4) these effects could be amplified by chronic stress due to early adversity and/or psychiatric comorbidity such as depression or anxiety in patients with chronic pain. Based on these considerations, ideas for much-needed interdisciplinary research are generated that could bridge the cognitive neurosciences with the fields of stress and chronic pain. Note that this perspective focusses specifically on associative learning processes. Nevertheless, instrumental (or operant) learning may be equally relevant in the pathophysiology and treatment of chronic pain (Flor, 2012; Gatzounis et al., 2012; Vlaeyen, 2015), but are beyond the scope.

## Chronic Pain and Stress

Chronic pain is a major and unresolved healthcare problem with significant individual as well as societal implications (Breivik et al., 2006). The broad relevance of psychosocial stress in the context of bio-psycho-social disease models of chronic pain is well-established (Lumley et al., 2011; Jennings et al., 2014). Chronic stress and psychiatric comorbidity constitute risk factors for the development and persistence of different types of chronic pain, including visceral pain such as in the irritable bowel syndrome or functional dyspepsia (Elsenbruch, 2011; van Oudenhove and Aziz, 2013), fibromyalgia syndrome (Schmidt-Wilcke and Clauw, 2011), chronic musculoskeletal pain (Finestone et al., 2008; Diatchenko et al., 2013) and migraine (Borsook et al., 2012). For many chronic pain conditions, non-pharmacological treatment approaches incorporate cognitive-behavioral techniques aiming to reduce stress, improve coping and/or ameliorate affective disturbances. In addition, central and/or peripheral stress mechanisms have been proposed as novel targets for therapeutics in the treatment of pain (McEwen and Kalia, 2010; Johnson and Greenwood-Van Meerveld, 2014; Nekovarova et al., 2014). In spite of this converging clinical evidence supporting the crucial importance of stress and altered stress systems in the etiology and pathophysiology of chronic pain (Borsook et al., 2012; Vachon-Presseau et al., 2013), the central mechanisms underlying interactions between stress (or stress mediators) and altered pain-related learning and memory processes remain unclear. Meanwhile, functional and structural brain alterations involved in the pathophysiology of chronic pain are increasingly well-characterized (Ossipov et al., 2010), and overlap with brain circuits involved in emotion regulation and stress (Baliki and Apkarian, 2015) as well as with regions mediating fear expression and recovery (Dejean et al., 2015).

## Pain-Related Fear

Learning to predict pain based on predictive internal or external cues can be considered a fundamental and highly adaptive process which allows the organism to evoke the above described range of complex responses aimed at self-protection. Pavlovian fear conditioning as a translational model in the neurosciences has proven highly fruitful to elucidate associative learning and extinction processes involving aversive stimuli (Milad and Quirk, 2012), including pain. Conceputally, as a result of contingent pairing of pain-predictive conditioned cues (CS^+^) with pain as unconditioned stimuli (US), differential conditioned responses (CR) in anticipation of pain can be evoked by presentation of the pain-predictive CS^+^ when compared to another cue that remains unpaired (CS^-^). These CR occur during pain anticipation and have been termed pain-related fear (or fear of pain), a concept that increasingly gains attention in the pain field (De Peuter et al., 2011; Vlaeyen, 2015; Zaman et al., 2015). In addition to pain-related fear as the most prominent response, pain-predictive CS demonstrably evoke a range of reactions, including increased arousal and selective attention, in line with the complex responses to acute pain described above. At the same time, cues signaling the absence of impeding pain (i.e., CS^-^ that remain unpaired with the US) appear to aquire a separate set of responses, in line with their role as safety signals. The relevance of a safety learning process as part conditioning with aversive US is not only supported by a recent brain imaging meta-analysis of human fear conditioing studies (Fullana et al., 2015), but also by experimental data in patients with chronic pain (Volders et al., 2012; Meulders et al., 2014; Icenhour et al., 2015b). While the role of deficient safety learning in the pathophysiology and treatment of chronic pain remains to be clarified, it is conceivable that these signals may further reinforce safety-seeking behavior as a key component of avoidance. Hence, it is likely that the interplay of conditioned pain-related danger and safety signals drives maladaptive avoidance behavior in chronic pain.

Whereas the relevance of classically-conditioned fear is well-established in the context of anxiety disorders (Milad and Quirk, 2012; Tovote et al., 2015), it is only beginning to be elucidated in the context of chronic pain. Fear conditioning studies have demonstrated altered fear learning in various patient groups with chronic pain, including fibromyalgia, chronic back pain, chronic tension-type headaches and irritable bowel syndrome as reviewed herein (Vlaeyen, 2015). In light of this converging evidence across diverse chronic pain conditions, it appears that altered acquisition of pain-related fear is clearly characteristic for chronic pain. Meanwhile, the specific contribution of conditioned pain-related fear to the pathophysiology of chronic pain remains an issue of ongoing research. Conceptually, pain-related fear has been embedded in fear avoidance models of chronic pain (Leeuw et al., 2007; den Hollander et al., 2010; De Peuter et al., 2011; Crombez et al., 2012). These models assume that a vicious circle of exaggerated pain-related fear and dysfunctional avoidance is maintained by emotional factors like increased anxiety as well as hypervigilance and pain catastrophizing. It has also been proposed that conditioning may lower pain thresholds (Williams and Rhudy, 2007) or promote sensitization (Overmier, 2002; Jensen et al., 2015) and thus contribute to hyperalgesia, impair perceptual discrimination acuity (Zaman et al., 2015), enhance fear generalization (Meulders et al., 2015) or interfere with normal habituation processes (Lowén et al., 2015). As part of a surgence in new research studies coming from within the pain field, innovative experimental paradigms have been introduced which implement different types of clinically-relevant painful stimuli as US and/or CS, including movement-related (e.g., Meulders and Vlaeyen, 2012) or visceral stimuli (e.g., Yágüez et al., 2005; Kattoor et al., 2013; Icenhour et al., 2015a) aiming to address pain-related fear in the context of different chronic pain conditions characterized by specific types of pain. Meanwhile, brain imaging studies addressing neural mechanisms in patients with chronic pain remain scarce (Labus et al., 2013; Icenhour et al., 2015b), and virtually nothing is known about the possible roles of affective comorbidity and stress in shaping disturbed acquisition and/or impaired extinction of pain-related fear.

## Extinction of Pain-Related Fear

Pain-related extinction processes and their underlying neural circuitry remain uncharted research territory, despite first evidence suggesting the efficacy of exposure-based interventions for chronic pain (Vlaeyen et al., 2002; Linton et al., 2008; Woods and Asmundson, 2008; Craske et al., 2011; Ljotsson et al., 2014) and behavioral data supporting impaired extinction in chronic low back pain (Schneider et al., 2004). Extinction—as a form of new inhibitory learning—can be studied using retrieval techniques including reinstatement and renewal paradigms, which have been applied in the context of explaining relapse and return of fear in anxiety disorders (Milad and Quirk, 2012; Vervliet et al., 2013a,b). The renewal effect describes the return of CR to the CS due to a change of the extinction context, while reinstatement is defined as the retrieval of an extinguished memory after unexpected and unpaired exposure to the US. Both techniques are considered important tools into the mechanisms of memory consolidation and reconsolidation, but have rarely been studied in humans with brain imaging techniques. It is readily conceivable, however, how both reinstatement and renewal phenomena could occur in everyday life of chronic pain patients, with possibly detrimental effects: For instance, an unexpected pain experience (i.e., reinstatement) or context change (i.e., renewal) may lead to the retrieval of previously conditioned pain-related fear, resulting in a resurgence of maladaptive avoidance behaviors. In our own experimental work in the field of visceral pain, we tested for reinstatement by presenting unpaired painful stimuli subsequent to an extinction phase. After two feasibility studies in healthy individuals (Kattoor et al., 2013; Gramsch et al., 2014), in a first study in patients with IBS we could show enhanced reactivation of previously extinguished conditioned fear as evidence by differential neural activation (Icenhour et al., 2015b). This calls for more work in patients with chronic pain in order to complement and extend fear conditioning with contextual manipulations, reviewed in Maren et al. (2013), especially using conditioning with clinically-relevant painful stimuli (Icenhour et al., 2015a).

## Peripheral and Central Mechanisms of Stress

Stress shapes many types of adaptive behaviors by interacting with emotional and cognitive central processes in order to facilitate adaptation. Given the well-known overlap between stress, affective disturbances, deficits in emotion regulation and chronic pain, integrating stress into future research into extinction learning appears important and in fact necessary.

The first, rapid response to acute stress is orchestrated by activation of the SNS resulting in a rapid release of adrenalin and noradrenalin. These hormones cannot easily pass the blood brain barrier, but stimulate the vagus nerve, which causes an increased noradrenergic tone in the brain by its action on regions in the brain stem (Roozendaal et al., 2009). These regions in turn influence several brain areas including the amygdala and prefrontal cortex, which are both crucially involved in the regulation of learned fear (Dejean et al., 2015; Maren and Holmes, 2015) as well as central pain processing (Ossipov et al., 2010; Baliki and Apkarian, 2015). A second, slower response is orchestrated by the HPA axis. Corticotrophin releasing hormone (CRH) is released from the paraventricular nucleus of the hypothalamus into the portal blood system. On reaching the pituitary, CRH stimulates adrenocorticotrophin (ACTH) release into the peripheral blood stream, which initiates the secretion of glucocorticoids (GCs; corticosterone in most laboratory animals, cortisol in humans) from the adrenal cortex (Joels and Baram, 2009). In contrast to the catecholamines, naturally occurring GCs (like all other steroid hormones) can pass the blood brain barrier. In the brain, GCs can act via two different intracellular receptors (sometimes referred to as type I or mineralocorticoid (MR) and type II or glucocorticoid (GR) receptor), which differ in their distribution and affinity (Joels et al., 2008). Moreover GCs can exert rapid non-genomic effects, via membrane bound MRs and GRs or via interaction with other neurotransmitter receptors (Joels et al., 2008). GCs can influence neuronal excitability, neuronal plasticity, dendritic remodeling and neurogenesis (Roozendaal et al., 2009; Maren and Holmes, 2015). Besides, multiple neurotransmitter systems like the cholinergic, noradrenergic, serotonergic and dopaminergic system are influenced by GCs (Joels et al., 2008). In sum, catecholamines and GCs can have rapid as well as delayed effects on the function and structure of the brain, and thereby affect emotion regulation, including the acquisition and extinction of learned fear. Of note, existing experimental data in humans primarily address effects of acute stress (or stress mediators, particularly GC) rather than chronic stress, as explained below.

## Effects of Acute Stress on Acquisition and Consolidation

**Figure 1:**
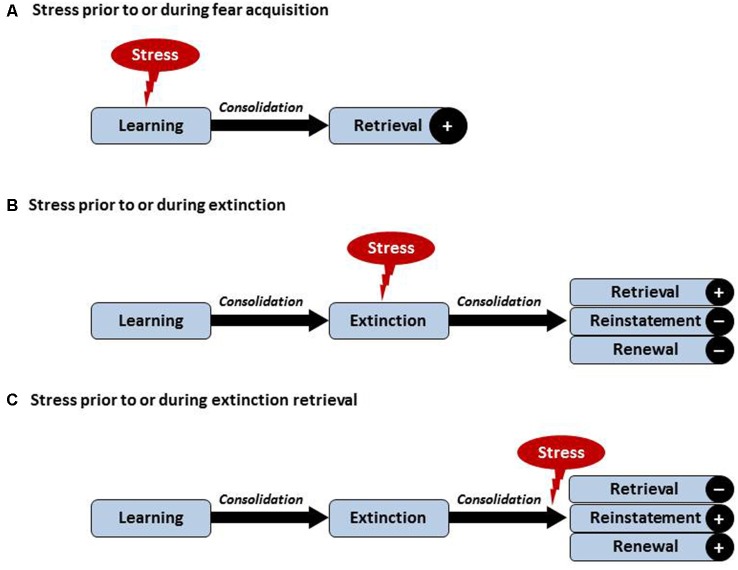
**Visualization of hypothesized effects of acute stress on learning and extinction of pain-related fear.** Postulated effects depend on the timing of acute stress in relation to acquisition and/or extinction. **(A)** Acute stress just before or during acquisition may facilitate the formation and/or consolidation of pain-related fear, reflected by greater retrieval of pain-related fear. **(B)** Acute stress just before or during extinction learning may improve consolidation of the extinction memory trace, possibly resulting in enhanced retrieval of the extinction memory and hence reduced reinstatement or renewal. **(C)** Post-extinction stress occurring just prior to or during extinction retrieval may impair the retrieval of the extinction memory and result in enhanced reinstatement or renewal effects. Note that chronic stress or affective comorbidity may differentially affect these processes involving central and peripheral mechanisms of the inter-connected stress and pain systems (not shown here, see text).

Effects of acute stress on learning and memory processes are demonstrably (learning) phase-dependent, requiring a careful separation of the processes underlying acquisition, consolidation, reconsolidation and retrieval (for an illustration, see Figure 1). In addition to aspects of timing, consequences of stress exposure and/or application of stress hormones appear to vary with the type of learning with possible differences between for example rather neutral declarative versus emotional learning tasks. Briefly, as reviewed in Raio and Phelps (2015) animal research on cued fear supports that stress exposure facilitates the acquisition and consolidation of cued fear. Similar findings exist in humans. It has been observed that pre-learning GC treatment (Buchanan and Lovallo, 2001; Kuhlmann and Wolf, 2006) or immediate post learning stress (Cahill et al., 2003; Preuß and Wolf, 2009) enhanced memory consolidation resulting in enhanced retrieval days to weeks later. In several studies, this effect was more pronounced for arousing material (Kuhlmann and Wolf, 2006; Smeets et al., 2008). The number of human studies explicitly addressing effects of stress on conditioned fear remains small with partially inconsistent findings, which may in part be due to sex differences (Merz et al., 2010, 2013) and divergent effects depending on the timing of stress with respect to the beginning of the acquisition (Hermans et al., 2014). Effects of acute stress on the acquisition of pain-related fear have not been studied thus far. We speculate that acute stress prior to or during acquisition of pain-related fear may facilitate memory formation and/or consolidation, which would result in greater retrieval of pain-related fear at later time points (see Figure 1A). This would be in line with clinical evidence that stress can lead to symptom reoccurrence in a wide range of disorders including stress-related and anxiety disorders (Wolf, 2008; Maren and Holmes, 2015).

## Effects of Acute Stress on Extinction

In the case of extinction learning and extinction retrieval, the fascinating question arises whether stress influences the original acquisition memory trace or the later developed inhibitory extinction memory trace. The influence of stress and the potential role of GCs on extinction have been investigated in rodents already in the 70’s of the last century (Bohus et al., 1970; Kovács et al., 1977). From these studies, the notion emerged that GCs facilitate extinction, an interpretation supported by more recent reports (Yang et al., 2006; Brinks et al., 2009). For example, Yang et al. (2006) demonstrated that intra amygdala infusion of a GR receptor agonist facilitated extinction, while blockage of GR production with metyrapone impaired extinction. Most of these previous studies did not test the long-term consequences (extinction retrieval) of the extinction manipulation (for a review, see Rodrigues et al., 2009).

In a series of studies involving healthy human volunteers, we have recently tested the impact of stress on extinction retrieval using a renewal paradigm (Hamacher-Dang et al., 2013; Merz et al., 2014). Results revealed that stress impaired extinction retrieval in a predictive learning task but impaired the retrieval of the original fear memory trace in a fear-conditioning task. These results are in line with the hypothesis that the more emotional memory trace is more heavily influenced by stress. With respect to extinction consolidation it could be demonstrated that post-extinction stress led to a more context-dependent extinction memory, which was associated with a more pronounced renewal effect (Hamacher-Dang et al., 2013, 2015). Taken together, the findings demonstrate that stress has a phase-dependent effect on extinction learning (see Figure 1) which is further modulated by the emotionality of the learning material and by the context in which the learning took place. Given the emotional components of pain and pain-related fear as an essentially emotional construct, this has interesting implications for the design of mechanistic studies aiming to test differential effects of stress on the retrieval of pain-related fear, as detailed below. The most important prediction that can be made is that acute stress during extinction learning may improve extinction learning resulting in greater retrieval of the extinction memory and thus reduced reinstatement or renewal (Figure 1B). On the other hand, stress just before or during extinction retrieval may result in reduced retrieval of the extinction memory trace and thus greater reinstatement or context-dependent renewal (see Figure 1C). These predictions could be tested using psychosocial stress models or administration of appropriate agonists of the HPA axis and/or the SNS.

## Reactivation, Reconsolidation and its Modification Through Stress Hormones

Recently interest in the phenomenon of reconsolidation has surged. Building on findings from the sixties (Misanin et al., 1968), Nader and colleagues were able to show in rodents that established fear memories become labile after reactivation (exposure to the CS) and have to reconsolidate again. Post reactivation protein synthesis inhibition completely erased the fear memory (for a review, see Nader and Hardt, 2009). For fear conditioning, similar results (i.e., impaired reconsolidation) could be obtained using a beta receptor blocker in rodents as well as humans (Kindt et al., 2009). Recent research from our group in humans has revealed that the stress hormone cortisol enhances fear reconsolidation (Meir Drexler et al., 2015). The enhancing effects of stress mediators on memory reconsolidation may in part explain the long lasting memories of aversive events. Each stressful reactivation will further strengthen the memory trace. Applying these findings to chronic pain, one could postulate that stress results in a reactivation of the pain-related memory trace and/or facilitates its reconsolidation, ultimately making the pain-related fear memory more permanent. This process may contribute to the maintenance of pain-related fear and maladaptive avoidance behavior as part of a vicious circle maintained by stress and fear. Furthermore, research into interactions between affective comorbidity, acute stress and memory processes may contribute to elucidating individual risk and vulnerability factors and neuropharmacological treatment options for chronic pain (Nekovarova et al., 2014).

## Conclusions and Future Directions

We propose that stress may be linked to impaired extinction and enhanced retrieval of pain-related fear in patients with chronic pain—hypotheses that are yet to be tested. In this much needed work, it is important to consider that the modulation of memory processes may differ depending on the type and duration of stress. A difference between acute and chronic stress is supported by data outside of the pain field: Early adversity as well as chronic stress has been linked to structural alterations in the brain causing a hyperactive amygdala and impaired prefrontal inhibition (Roozendaal et al., 2009). These alterations could underlie the extinction impairments observed in several mental disorders (Maren and Holmes, 2015), which is interesting in the context of chronic pain given the high comorbidity between chronic pain and affective disorders. Finally, effects of acute stress on pain-related memory retrieval may be fundamentally different in normals and patients with chronic pain. While stress-induced effects in normals are adaptive, they may be altered and in fact maladaptive and in patients with chronic pain. For example in patients with PTSD, cortisol enhanced rather than impaired memory retrieval [for review, see Wingenfeld and Wolf (2015)]. Another future research area is to address if and to what extent stress may affect overgeneralization, as recently shown in patients with fibromyalgia (Meulders et al., 2015) and/or perceptual discrimination (Zaman et al., 2015). Clearly, more patient-oriented experimental work is needed to disentangle the complex interactions between acute and chronic stress and different pain-related memory processes encompassing extinction, extinction retrieval and memory consolidation and reconsolidation. Ultimately, this work could be the basis for an improvment of existing treatment approaches for patients with chronic pain, who benefit from exposure-based interventions. Studies in anxiety patients have observed that cortisol enhances the success of extinction-based therapies in patients with fear of heights (de Quervain et al., 2011) as well as in spider phobics (Soravia et al., 2014), presumably by boosting extinction consolidation. Although effects of acute stress or acute GR administration on extinction of pain-related fear have not been tested humans, this could be a promising endeavor for patients with chronic pain.

## Funding

Both authors are members of the research unit “Extinction Learning: Behavioural, Neural and Clinical Mechanisms” funded by the German Research Foundation (DFG, FOR 1581).

## Conflict of Interest Statement

The authors declare that the research was conducted in the absence of any commercial or financial relationships that could be construed as a potential conflict of interest.
